# A Novel Monitoring Approach for Train Tracking and Incursion Detection in Underground Structures Based on Ultra-Weak FBG Sensing Array

**DOI:** 10.3390/s19122666

**Published:** 2019-06-13

**Authors:** Qiuming Nan, Sheng Li, Yiqiang Yao, Zhengying Li, Honghai Wang, Lixing Wang, Lizhi Sun

**Affiliations:** 1 National Engineering Laboratory for Fiber Optic Sensing Technology, Wuhan University of Technology, Wuhan 430070, China; nqm0723@whut.edu.cn (Q.N.); wanghh@whut.edu.cn (H.W.); lxwang@whut.edu.cn (L.W.); 2 School of Information Engineering, Wuhan University of Technology, Wuhan 430070, China; yqyao@whut.edu.cn (Y.Y.); zhyli@whut.edu.cn (Z.L.); 3 Department of Civil and Environmental Engineering, University of California, Irvine, CA 92697-2175, USA; lsun@uci.edu

**Keywords:** underground structure safety, train tracking, incursion detection, ultra-weak FBG, distributed vibration, dynamic measurement

## Abstract

Tracking operating trains and identifying illegal intruders are two important and critical issues in subway safety management. One challenge is to find a reliable methodology that would enable these two needs to be addressed with high sensitivity and spatial resolution over a long-distance range. This paper proposes a novel monitoring approach based on distributed vibration, which is suitable for both train tracking and incursion detection. For an actual subway system, ultra-weak fiber Bragg grating (FBG) sensing technology was applied to collect the distributed vibration responses from moving trains and intruders. The monitoring data from the subway operation stage were directly utilized to evaluate the feasibility of the proposed method for tracking trains. Moreover, a field simulation experiment was performed to validate the possibility of detecting human intrusion. The results showed that the diagonal signal pattern in the distributed vibration response can be used to reveal the location and speed of the moving loads (e.g., train and intruders). Other train parameters, such as length and the number of compartments, can also be obtained from the vibration responses through cross-correlation and envelope processing. Experimental results in the time and frequency domains within the selected intrusion range indicated that the proposed method can distinguish designed intrusion cases in terms of strength and mode.

## 1. Introduction

During the last few decades, the construction of urban subways has developed rapidly worldwide and particularly in China. Aiming to ensure the operational safety of subways, a wide range of research effort has been undertaken in the fields of the subway fires [1,2,3], structural safety [4,5,6,7], and perimeter invasion [8,9,10,11]. Among these three fields, structural safety monitoring and perimeter intrusion detection are of more concern than fire monitoring due to the diversification of demand. For long-distance monitoring needs, especially for subway tunnels, distributed fiber-optic sensing technology has been widely recognized as the most promising means of addressing complex needs due to its advantages of large-scale monitoring, high sensitivity, and multiplexing capabilities [12]. For instance, the safety monitoring of subway structures based on Brillouin optical time domain reflectometry (BOTDR) technology [13] has been reported [14,15]. In addition to BOTDR-based static measurement, distributed fiber-optic sensors for dynamic measurement [16], especially distributed acoustic sensing (DAS) technology [17,18] have been another research hotspot. The use of DAS technology to ensure the safety of subway operations has also attracted widespread attention for both engineers and researchers.

As one of the main concerns for ensuring the operational safety of the subway, tracking e subway trains occupies an important position in the train operation control system; tracking is directly related to train safety and affects the transportation efficiency of the rail transit. In addition to tracking trains in operation, positioning illegal intruders and preventing the risk caused by such intrusion events—which usually occur during the subway outage periods—is another issue worth noting. For the former, Peng et al. [19] reviewed the shortcomings of conventional train positioning techniques and pioneered investigation of the feasibility of train positioning and speed monitoring through Φ-OTDR technology, in which the spatial resolution of the common optic fiber reaches 20 m. He et al. [20] reported a DAS-based method for condition monitoring of the running train, claiming that the train positioning error was less than 20 m. For human intrusion, Catalano et al. [21,22] reported an incursion detection system for railway security using two types of fiber Bragg grating (FBG) sensors, which is apparently only applicable to a limited protection area due to the restricted multiplexing capacity of FBG. In addition, He et al. [23] presented research on railway perimeter safety based on DAS technology, which has a spatial resolution of only 10 m in the reported application scenarios.

Obviously, the dynamic measurement techniques based on DAS provide feasible detection methods for train tracking and human intrusion. Yet, there are still few research efforts on integrated methods for both train tracking and intrusion detection. Compared with DAS technology using common optic fiber, ultra-weak FBG arrays based on the draw tower [24,25] using sensing optic fiber integrates both advantages of fiber optic point sensors and distributed sensors. This technology is a new way to achieve high-precision, fast and wide coverage distributed measurement. Previous research around this technology has focused more on monitoring strain, temperature or strain-based deformation for the object of interest [26,27,28]. In addition, a multi-parameter measurement system based on an ultra-weak FBG array with sensitive material was proposed in [29]. However, all this research is still limited to static indicators. In fact, ultra-weak FBG array is also adept at performing dynamic monitoring [30,31] in addition to the above positive characteristics usually witnessed in static measurement. The reports in [16,32,33,34] revealed that the ultra-weak FBG array can not only be used for both static and dynamic measurements, but also has a higher signal-to-noise ratio (SNR) than that of DAS sensors. It is known that higher SNR often leads to better sensing performances such as higher measurement accuracy, faster response time and simpler detection circuit. Therefore, the ultra-weak FBG array is more suitable than DAS when dealing with distributed vibration and other scenarios requiring high-speed measurement.

To eliminate the need for two separate systems, improve measurement efficiency and reduce overall cost, this paper explored the feasibility of addressing train tracking and human intrusion in subway systems using distributed vibration measurement based on the ultra-weak FBG sensing array. The experimental results of identifying running trains and intruders in an actual subway are reported. The sensing and monitoring principles make up the second part of this paper, followed by the details of the design and field arrangement used to validate the proposed method. Finally, the effectiveness on tracking and detecting the objectives of interest is discussed based on the experimental results represented by the responses of distributed vibration of the ultra-weak FBG array.

## 2. Sensing and Monitoring Principles

**Figure 1:**
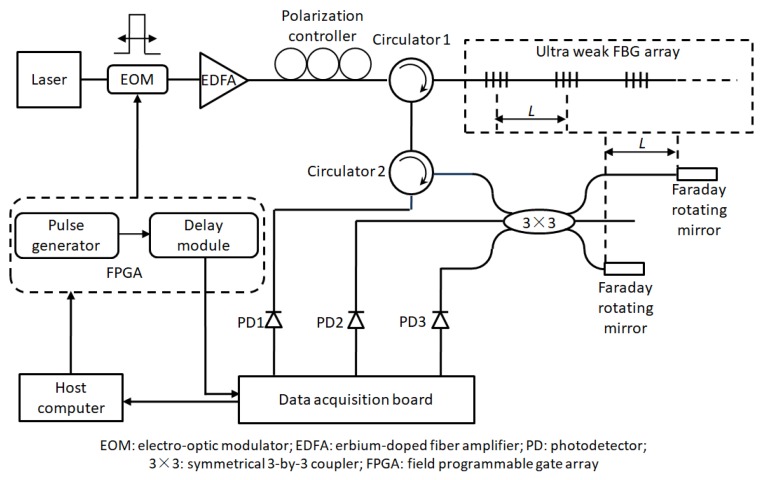
Sensing principle of distributed vibration detection-based on ultra-weak FBG array.

Figure 1 illustrates the distributed vibration sensing principle used to detect distributed vibration generated by moving loads, such as trains, intruders and so on. The phenomenon of light interference caused by the reflection signals of adjacent two ultra-weak FBGs is used to detect the vibration of the object of interest. Here, the ultra-weak FBG [35] is regarded as a mirror, and *L* represents the distance that causes light interference. In order to ensure a stable optical interference effect and overcome the occurrence of optical interference failure due to the difficulty of matching adjacent ultra-weak FBG caused by, for example, temperature variation drift, ultra-weak FBG in the array uses 3 nm wideband FBG. In addition, since the temperature changes slowly with respect to vibration, the temperature influence is ignored in the demodulation process of the vibration. The spatial resolution of the distributed vibration along the sensing optic fiber is typically determined by the parameter *L*. The sensitivity and the frequency response of the vibration signal measured by the strain-induced phase variation between two ultra-weak FBGs are improved by the interferometer. Here, Faraday rotating mirrors are utilized in the demodulation process of the ultra-weak FBG array to suppress the polarization effect. Moreover, the 3-by-3 coupler phase demodulation algorithm is used to reconstruct the time domain signal, and restore the phase information of the vibration signal, through which the interrogation of the vibration frequency and amplitude can be realized. Further, the optical time domain reflectometry technique is utilized to achieve vibration localization, and therefore, increasing the length of the optical cable will prolong the sampling interval of the vibration signal and reduce the response bandwidth of the system.

**Figure 2:**
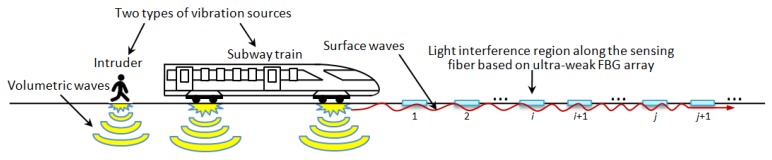
Monitoring principle of capturing the two types of moving loads of interest based on distributed vibration.

The high sensitivity of large-scale ultra-weak FBGs and the corresponding demodulation system of high speed [36] make the sensing optic fiber particularly suitable for locating structural vibration excited by moving loads occurring within a long-distance range. In addition, the previous study [37] revealed the repeatability of such a sensor represented by strain is around 3.41 nano epsilon. When dealing with train tracking and intrusion detection, either the train or intruder movement can be regarded as a vibration source. Owing to such excitation, the surface waves propagate omni-directionally on the ground. Because the surface wave couples to the track bed and rail track, distributed sensing optic fiber mounted beside the rail track along the subway can detect the vibration generated by a passing train or human footsteps (see Figure 2). The light interference region indicated by the address of ultra-weak FBG can be interrogated with the time- and wavelength-division multiplexing method [38,39], causing each known light interference region along the sensing optic fiber to have a determinate correspondence with the mileage. This also indicates that querying the interference region generated by the distributed vibration excitation is a viable way to track or detect the moving load of interest.

Moreover, the speed of the train or intruder can be determined through the τ lag time described in cross-correlation Equation (1) and the known distance between regions *i* and *j* as depicted in Figure 2.

(1) $$R_{s_{i}s_{j}}(\tau )=\underset{T\to \infty }{\lim}\frac{1}{T}\int_{0}^{T}S_{i}(t)S_{j}(t+\tau )$$

where $S_{i}(t)$ and $S_{j}(t)$ represent the vibration response at light interference regions *i* and *j*, respectively. The lag time τ is equal to the duration from the regions *i* to *j*. Further, through draw-tower grating preparation with five-meter equidistance between adjoining FBGs along the sensing optic fiber, the spatial resolution of the sensing optic fiber discussed in this paper enabled better positioning accuracy of tracking the train and intruder than that of the above-mentioned reports in actual engineering practice.

## 3. Design and Field Arrangement for the Experiments

### 3.1. Engineering Background of the Trial

**Figure 3:**
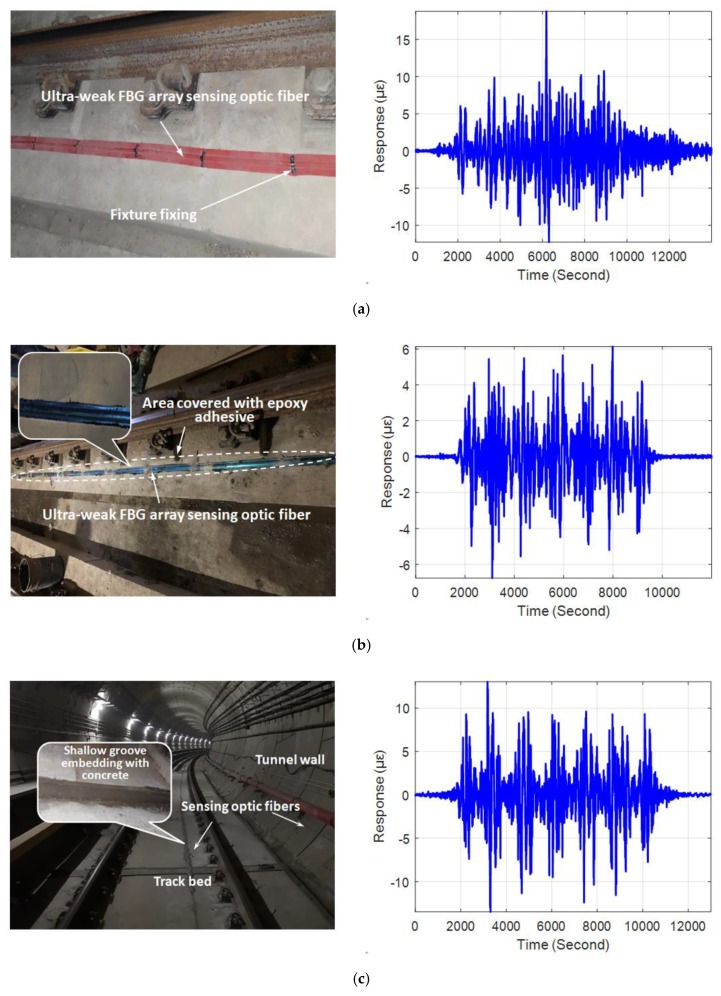
Methods of affixing distributed vibration sensing optic fibers and typical vibration response induced by train: (**a**) fixture fixing, (**b**) epoxy adhesive, and (**c**) shallow groove embedding.

An actual tunnel structure (Wuhan Metro Line 7) was used in this study. Before the operation of the subway, the ultra-weak FBG sensing optic fiber with armored protection using a layer-stranding structure with a loose tube was installed on structure surfaces of the selected tunnel segments, as in the actual layout shown in Figure 3. Here, the research on identifying the two types of moving loads was primarily based on the track bed response. To better obtain the vibration response of the track bed, three methods for fixing the sensing optic fiber to the track bed were tried to evaluate the suppression effect of the disturbance vibration—namely, fixture fixing, epoxy adhesive and shallow groove embedding. The typical vibration responses of a monitoring zone induced by passing trains in each fixing method are shown on the right side of Figure 3. It can be seen that as the coupling constraint between the sensing array and the track bed increased, the amplitude symmetry of the vibration response improved, and the peak regularity of the vibration response associated with train excitation became clear. Therefore, shallow groove embedding was adopted to affix the sensing array to secure a better signal output.

**Figure 4:**
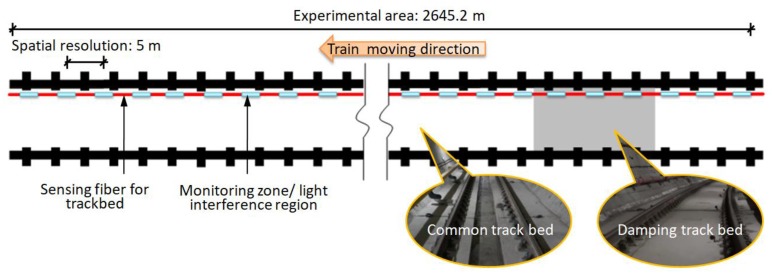
Schematic diagram of experimental area covering different track bed structures.

The designed monitoring system can guarantee five kilometers array length and reach multiplexing capacity of 1000 ultra-weak FBGs. As shown in the schematic diagram in Figure 4, the experimental area covered three underground stations with a total length of nearly three kilometers. Due to the spatial resolution of the sensing optic fiber and the specific layout of the tunnel structure, more than 500 vibration regions labeled #1 to #515 along the track bed can be distinguished based on the interrogated address of the light interference. It can be seen from the right side of Figure 4 that in addition to the common track bed structure, the damping track bed was also included in the experimental area. During the trial, the real-time vibration responses with a 1 kHz sampling rate were fully transmitted back to the platform monitoring center and processed by the demodulator and servers. Since the ultra-weak FBG array was fabricated simultaneously in the optic fiber drawing, there was no additional splice in the sensing optic fiber equipped with armored protection, except for the pigtail that needed to be connected to the demodulation instrument.

### 3.2. Train Tracking Trial

Because the subway line has already been in operation, the distributed vibration responses of the experimental area caused by the train were automatically collected and directly taken as the raw data for the trial. In addition to observing the response caused by the train traveling in the subway tunnel monitored by the sensing optic fiber, the test discussed the identifiability of the sensing optic fiber to the train moving in the opposite direction in the adjacent tunnel. Based on the single point response and overall distribution characteristics, the detection capabilities of the following indicators were discussed in turn: the speed and position of the train, the response difference between the common and damping tracks, and other parameters of the train.

### 3.3. Intruder Detection Trial

**Figure 5:**
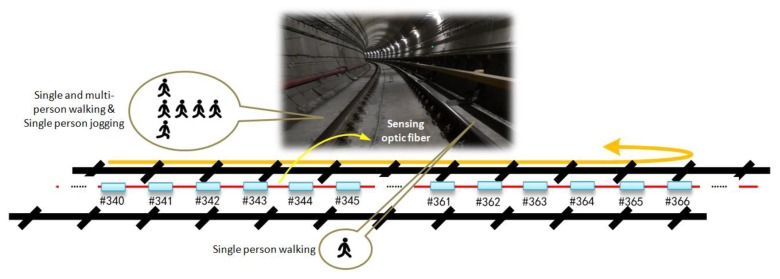
Simulated human intrusion scenarios along the rail track in the selected tunnel area.

To ensure the safe operation of the subway in the following day, various manual inspections are usually carried out at the subway outage in the early hours of the morning. We conducted the incursion test at this inspection window; this is also the period in which illegal intrusion usually occurs. Based on the specific circumstances and various scheduled tasks, a range in the area of the damping track bed was approved for performing multiple sets of simulated intrusion tests. To minimize cross interference from other simultaneous inspections, the trials were primarily concentrated within a 130 m range of the selected tunnel area. As shown in Figure 5, the trials simulated single and multi-person intrusion scenarios and considered the intrusion patterns of walking and jogging. In each trial, the participant in the simulation test made a round trip within the designed intrusion area.

## 4. Results and Discussion

This section reports the characteristics of distributed vibration responses under operating train and simulated incursion conditions, respectively. Feasibility, based on the proposed comprehensive approach concerning train tracking and detecting incursion, was investigated and is discussed. All the following analyses were based on the original output of the distributed vibration responses with no additional techniques adopted to improve the data quality.

### 4.1. Distributed Vibration Responses under Load of Passing Train

**Figure 6:**
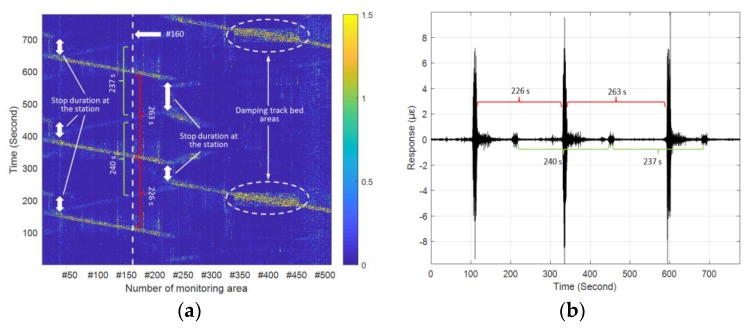
(**a**) Vibration intensity versus space and time under operating train; (**b**) original time series of vibrations of monitoring zone #160.

Figure 6a depicts a typical visualization relationship between the structural vibration intensity and the space and time. Here, the vibration intensity was represented by color of the figure. A waterfall diagram such as Figure 6a can be used to help analyze the train’s running direction, speed change and arrival or departure interval. In the tunnel where the sensing optic fiber arrays were deployed, the train entered the experimental area from #500 monitoring zone in Figure 6a. In this case, a moving train appeared as a diagonal signal pattern where the slope depended on the speed. Here, the diagonal signal pattern highlighted the characteristics of the distributed vibration response caused by the passing train within the monitoring range. Three complete sets of such diagonal signal patterns can be clearly seen in the left part of Figure 6a. Moreover, vibrations generated by moving trains in the opposite direction in the adjacent tunnel were simultaneously detected by the sensing optic fiber, although the vibration intensity was somewhat weak. Further, the process of the train stopping at the station between the diagonal signal patterns can also be observed in the figure. For monitoring zones #250–#500, the region range of the damping track bed structure, can be clearly identified based on the height changes (along the time axis) of the diagonal signal pattern. Due to the large design distance between the tunnel where the experimental areas #250–#500 were located and the adjacent tunnel, the vibration transmitted from the adjacent tunnel becomes invisible in the right part of Figure 6a.

**Figure 7:**
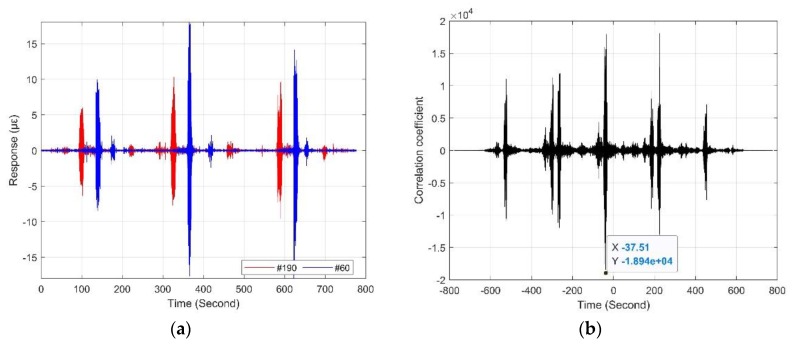
(**a**) Original time series of vibrations at zones #60 and #190; (**b**) time lag between zones #60 and #190.

Figure 6b presents a complete vibration response of one monitoring zone in Figure 6a, which quantifies the difference in vibration intensity of the sensing optic fiber caused by the train moving in two adjacent tunnels. In addition, the time interval of the two adjacent trains was approximately four minutes as observed in Figure 6, which was consistent with the planned subway operating timetable. Moreover, compared with the report based on Φ-OTDR [18], the method using sensing optic fiber in this paper did not require the multiple averaging technique to improve the SNR of the original time series of vibrations, which was more efficient for providing location information of the detected object. Therefore, the responses of any two different monitoring zones could be used to determine the train speed. For instance, Figure 7a shows the intensity projections of the two measurement areas on the time axis of Figure 6a, the cross-correlation analysis (see Figure 7b) of the vibration sequences (see Figure 7a) of the two monitoring zones at 650 m apart indicated that the train took 37.51 s to pass through the two selected zones. In this way, the train speed of 62 km/h can be obtained. Moreover, it was found that the amplitudes of these two monitoring areas were different, although the sensing optic fiber and its fixation method were consistent. The reason for this was primarily due to uneven geological properties of the underground structure along the mileage direction of the tunnel and different design curvature along the tunnel line, and the structural stiffness of the shield segments.

**Figure 8:**
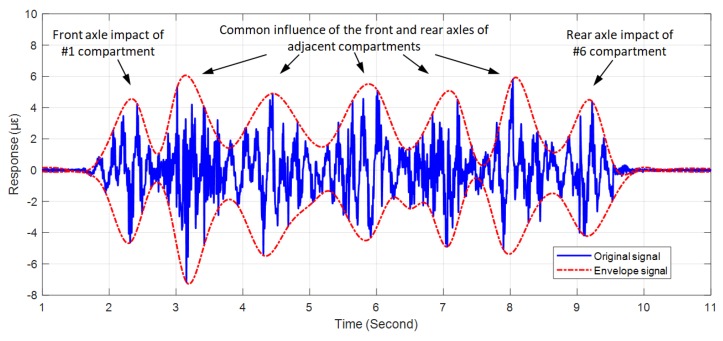
Original time series of vibration and corresponding envelopes during the passage of the train through one monitoring zone.

In addition, the vibration response obtained by a particular monitoring area during the passage of the train can reflect some geometric parameters of the train, such as its length and the number of compartments. The former, length, can be estimated by the calculated speed and the known height changes of the diagonal signal pattern. The latter, number of compartments, can be revealed by the number of peaks or valleys of the envelope signal. Figure 8 shows a typical vibration response of a monitoring zone between #60 and #190 during train passage. The vibration response lasted for about 8.5 s, corresponding to the height change of the diagonal pattern shown in Figure 6a. Based on the obtained average speed of 62 km/h, the calculated train length of 146 m was close to the actual known 142 m. Also, seven envelope peaks and valleys can be recognized in Figure 8 by envelope processing. This envelope result agreed well with the axle impact of the six train compartments.

### 4.2. Distributed Vibration Response under Footsteps of Intruder

**Figure 9:**
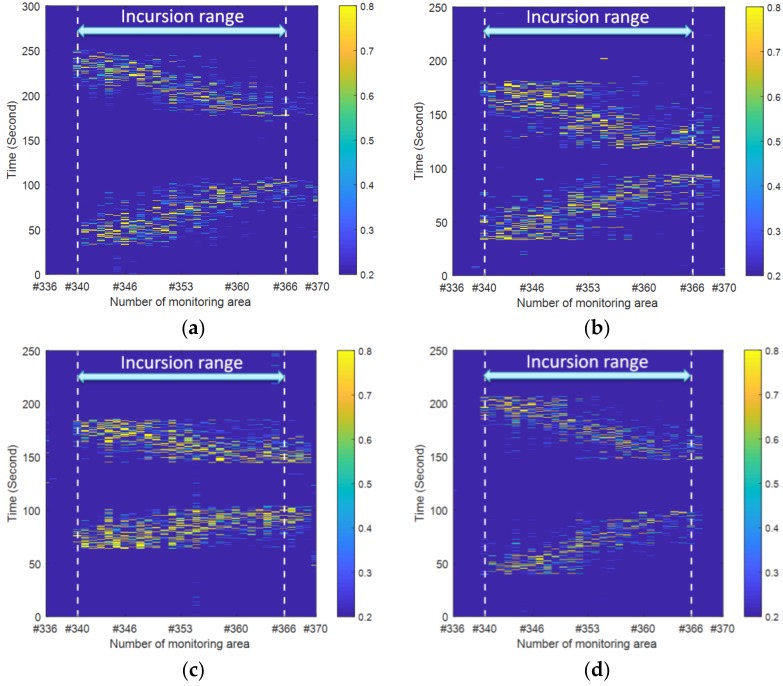
Vibration intensity versus space and time under different intrusion scenarios: (**a**) single person walking; (**b**) four people walking; (**c**) single person jogging; (**d**) single person walking along the other side.

**Figure 10:**
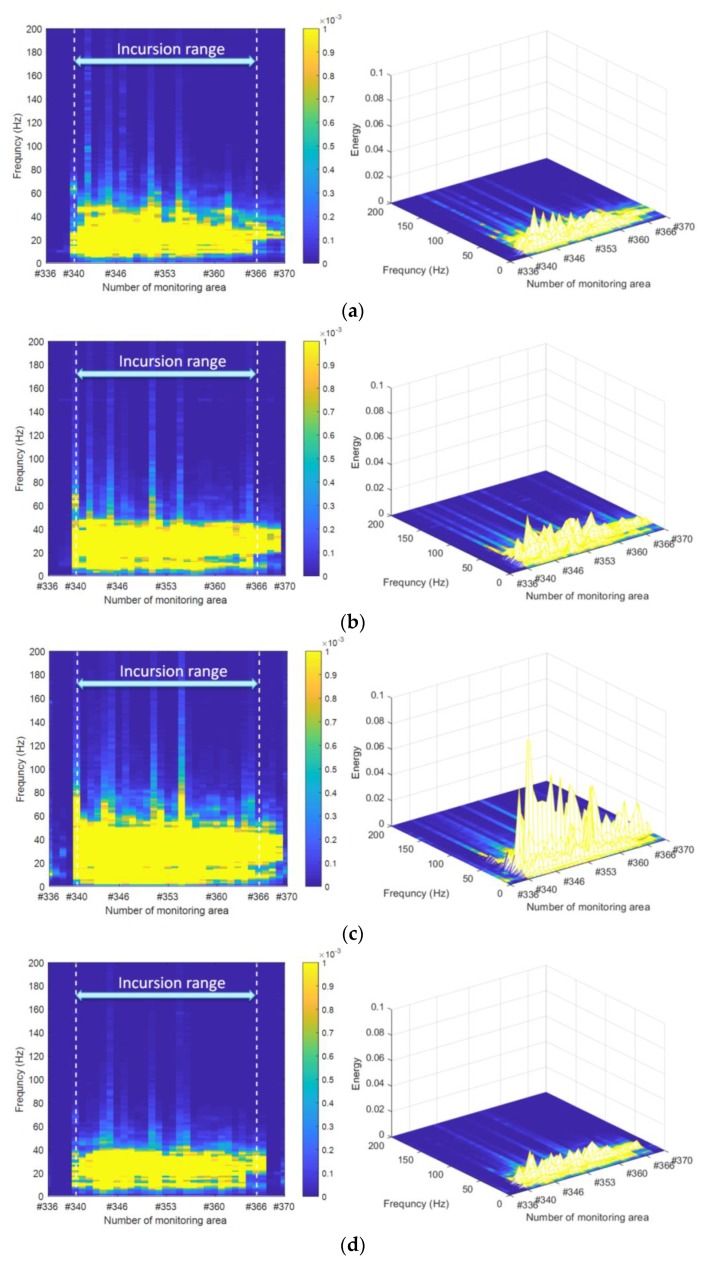
Vibration intensity versus space and frequency under different intrusion scenarios: (**a**) single person walking; (**b**) four people walking; (**c**) single person jogging; (**d**) single person walking along the other side.

Figure 9 and Figure 10 show the results of the designed human intrusion in the time and frequency domains, respectively. Moreover, the experimental results in the frequency domain for each of the designed cases are depicted two-dimensionally (left) and three-dimensionally (right) in Figure 10. Figure 9 reveals that significant distributed vibration responses generated by walking or jogging as defined in Figure 5 can be detected within the incursion range under both sides of the track. In addition, two diagonal signal patterns in the opposite direction further verified the simulated incursion process represented by round-trip walking or jogging. Furthermore, based on the different slope pattern caused by different speeds of the intruder, it was easy to distinguish the intrusion mode of jogging shown in Figure 9c from the other three intrusion modes of walking. This result was consistent with Figure 10 and was more pronounced in the frequency domain, where the intrusion caused by the jogging shown in Figure 10c led to the maximum fluctuation of the vibration intensity.

**Figure 11:**
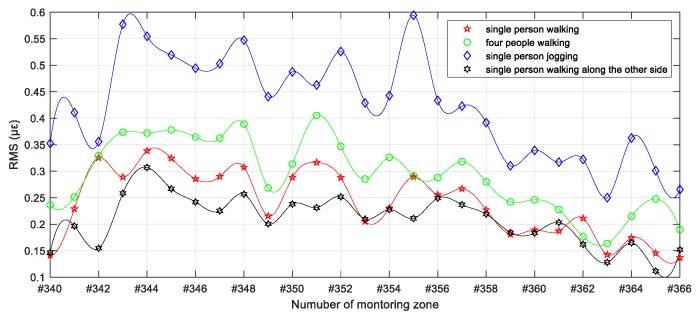
Fitting distribution of effective values of vibration response of incursion range under different simulated intrusion cases.

**Figure 12:**
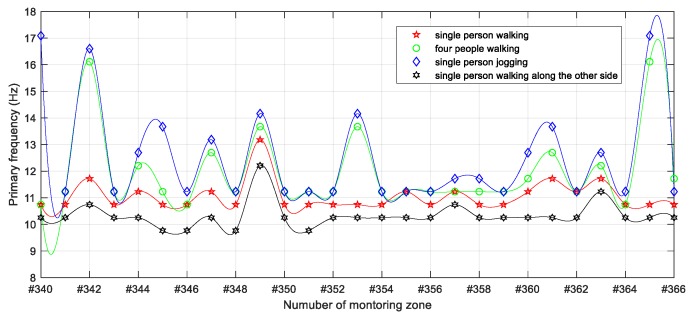
Fitting distribution of primary frequencies of vibration response of incursion range under different simulated intrusion cases.

To further quantify the different intrusion patterns reflected in Figure 9, the effective value represented by the root-mean-square (RMS) of the vibration response signal for each monitoring zone within the intrusion range in the whole test process was calculated and is shown in Figure 11. Here, the effects caused by personnel in the round-trip process outside the incursion range were not involved in the evaluation. As can be seen from the overall distribution of Figure 11, in addition to the significant difference between jogging and walking intrusion, the dynamic distributed vibration response can distinguish between single and multi-person walking intrusions. Moreover, subtle differences of vibration distribution caused by a single pedestrian intrusion at different distances from the sensing optic fiber can also be observed. Furthermore, Figure 12 quantifies the results represented in Figure 10 by the overall distribution of primary frequency. Here, the frequency value corresponding to the maximum energy of each column represented in Figure 10 was selected as the primary frequency for each monitoring zone. Table 1 further provides the statistical results for the four types of intrusion cases for Figure 12, where cases 1–4 represent a single person walking, four people walking, single person jogging and single person walking along the other side, respectively.

Since the dynamic characteristics of the structure within the incursion range and the forced vibration mode related to intrusion load frequency and type were different, it can be seen from Figure 12 that the primary frequencies excited by the simulated intrusion were different in the incursion range. However, the similarity of the distribution features in the different intrusion cases shown in Figure 12 can still be observed. That is, the distribution patterns of cases 1 and 4 were closer due to single pedestrian intrusion, while cases 2 and 3 exhibited more broad frequency information under stronger and more complex excitations. The calculated result of the mode values of primary frequency under each case shown in Table 1 further verified this opinion. In addition to the distribution feature, different maximum primary frequencies shown in Table 1, and varied fluctuation strength in Figure 12, also contributed to distinguishing different simulated human intrusions based on the frequency domain results of dynamic distributed sensing of ultra-weak FBG.

## 5. Conclusions

This study reported an integrated monitoring technology used for ensuring the safety of subway operation, which verified that dynamic distributed measurement based on ultra-weak FBG was a feasible method, suitable for both train tracking and human intrusion detection in an actual engineering application. The analysis based on subway operation monitoring illustrated that the location, speed, length, and number of train compartments could be determined through the vibration responses and distribution on the track bed. Moreover, the results of the simulated human intrusion performed in the damping track bed area during the subway outage period demonstrated that the sensing optic fiber had the potential to distinguish the strength and pattern of intruders. In view of the available test time and experimental range, the simulated cases of human intrusion were relatively limited and the detection effectiveness in the common track bed was not taken into account; this seems to be less than complete and deserves further attention when conditions permit. However, the advantages determined by the high SNR of ultra-weak FBG, when compared to other distributed sensing technologies based on common optic fiber, make us believe that the proposed method is promising for recovering and identifying signals in more complex modes.

## Figures and Tables

**Table 1:** Primary frequency characteristics of the different intrusion cases within the experimental area (unit: Hz).

Comparisons	Case 1	Case 2	Case 3	Case 4
Maximum	13.18	16.11	17.09	12.21
Mode ^1^	10.74	11.23	11.23	10.25

^1^ The most frequent primary frequency in the monitoring zones of the incursion range.
